# Enoxacin ameliorates polycystic ovary syndrome by promoting the browning of white adipose tissue and restoring gut dysbiosis

**DOI:** 10.3389/fphar.2022.978019

**Published:** 2022-09-06

**Authors:** Wanlong Zhu, Liya Fu, Changjing Xu, Ke Peng, Yuanzhi Liu, Hui Tang, Yilan Huang, Xuping Yang

**Affiliations:** ^1^ Department of Pharmacy, The Affiliated Hospital of Southwest Medical University, Luzhou, China; ^2^ School of Pharmacy, Southwest Medical University, Luzhou, China

**Keywords:** enoxacin, PCOS, insulin resistance, white fat browning, intestinal microbiome

## Abstract

Polycystic ovary syndrome (PCOS) is a complex endocrine disorder syndrome characterized by polycystic ovary, ovulation disorder and hyperandrogenemia, and is often accompanied by metabolic disorders. Enoxacin has been reported to protect against diet-induced obesity and insulin resistance by promoting fat thermogenesis. However, the function of enoxacin in PCOS remains unknown. This study aimed to investigate the impact of the enoxacin on the regulation of PCOS mouse model induced by dehydroepiandrosterone (DHEA). Here, we found that reproductive endocrine disorder, glucose intolerance, and ovarian dysfunction in PCOS mice induced by DHEA were attenuated by enoxacin treatment. Mechanistically, we identified that enoxacin can promote white fat browning and improve metabolic disorders, thus ameliorating DHEA-induced reproductive dysfunction. Moreover, these beneficial effects might be associated with the restoration of gut dysbiosis. These findings provide a novel therapeutic target for enoxacin in the treatment of PCOS.

## Graphical Abstract

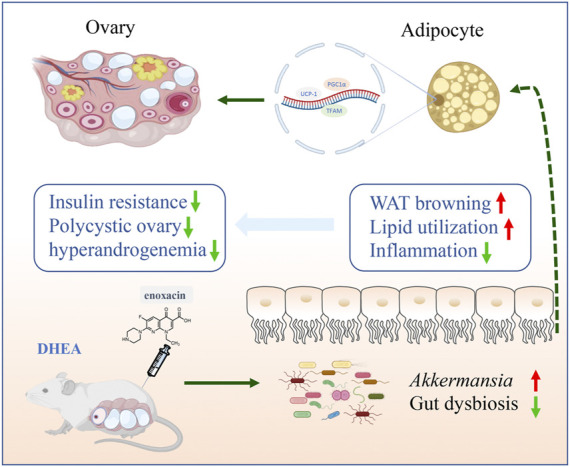

## Introduction

Polycystic ovary syndrome (PCOS) is a metabolic syndrome characterized by polycystic ovary, ovulation disorder and hyperandrogenemia (Azziz et al., 2016). It affects about 5%–20% of women of reproductive age, and the underlying molecular mechanism remains unknown (Haudum et al., 2020; Stener-Victorin and Deng, 2021). Insulin resistance (IR) is considered to be an important pathophysiological mechanism of PCOS, which directly or indirectly stimulate androgen secretion by ovarian theca cells, ultimately resulting in increased androgen (Teede et al., 2007; Wu et al., 2014). Given the crucial role of IR in PCOS, therapeutic interventions with insulin-sensitizer are usually used to manage PCOS (Escobar-Morreale, 2018). Adipose tissue is one of the major targets for insulin, and an increasing number of evidence indicate that the function and morphology of adipose tissue are aberrant in PCOS patients, androgen excess can cause adipocytes hypertrophy, and both adipose tissue hypertrophy and hyperandrogenism are related to IR (Goodarzi et al., 2011; Barber and Franks, 2013). White fat browning by small-molecule compound or transplantation of brown adipose tissue reversed ovarian dysfunction and IR in PCOS (Holmes, 2016; Qi et al., 2020), and these findings provide novel insights into the treatment of PCOS. Recently, it has been shown that fat metabolism can be regulated by gut microbiota, and gut dysbiosis is associated with the development of obesity and its related metabolic disorders (Cani et al., 2019).

Enoxacin is a broad-spectrum fluoroquinolone antibacterial agent that has been officially approved by the U.S. Food and Drug Administration for treating urinary tract infections (Rocha et al., 2020). In addition, as a stimulator of RNA interference and microRNA activity, enoxacin also displays some biological effects on tumor growth or bone disease (Melo et al., 2011; Cornaz-Buros et al., 2014; Liu et al., 2014; Zhao et al., 2022). A recent study revealed that enoxacin can increase adipose tissue thermogenesis and protect mice from dietary obesity and IR, which is achieved by a reduction of miR-34a-5p expression and upregulation of its downstream target genes (Rocha et al., 2020). Given the essential role of adipose tissue in the development of PCOS, we suspect that enoxacin could alleviate reproductive disorders by improving adipose function in PCOS. Here, we identified that enoxacin can promote beige adipogenesis and improve metabolic disorders in mice models of PCOS, thus ameliorating reproductive dysfunction induced by DHEA. Moreover, these effects might be associated with the restoration of gut dysbiosis. These observations suggest a potential new treatment drug for PCOS and a novel way of enoxacin to ameliorate energy metabolism.

## Materials and methods

### Animal and experimental design

In this study, female pre-puberty C57BL/6 mice (21 days old) were purchased from Beijing HFK bioscience Co. Ltd. (Beijing, China). All the mice were maintained in a pathogen-free environment (3–4 mice per cage), with 12-h light/dark cycle (lights on at 7:00 a.m.), under conditions of controlled temperature (23 ± 2°C) and humidity (50% ± 5%). All animal care and experimental procedures complied with the National Institutes of Health guidelines and were approved by the Animal Ethics Committee of the Garment Hospital of Southwest Medical University. Animals in the DHEA group were injected daily with DHEA (D4000; Sigma-Aldrich; 6 mg per 100 g, dissolved in 0.2 ml of sesame oil) subcutaneously. Enoxacin (100 mg/ml, MB1658-S, Meilun, Dalian) was initially dissolved in 1 M NaOH solution to a concentration of 312 mM and then diluted ×100 in phosphate-buffered saline (PBS) for injection. Vehicle was 10 mM NaOH in PBS.

In the intervention study, all mice were acclimated by placing them on a control chow diet administered *ad libitum* for 1 week and then randomly divided into five groups, three chow diet groups and two high-fat diet (HFD) groups: control group (Ctrl) that received normal chow diet with sesame oil for 3 weeks and another 3 weeks of PBS; DHEA + Vehicle (DHEA + Veh) group that received chow diet with DHEA for 3 weeks and another 3 weeks of PBS, DHEA + enoxacin (DHEA + EX) group that received chow diet with DHEA for 3 weeks and another 3 weeks of enoxacin (100 mg/kg/day) by intraperitoneal injection, HFD + DHEA + Vehicle (HFD + DHEA + Veh) group that received HFD with DHEA for 3 weeks and another 3 weeks of PBS, HFD + DHEA + enoxacin (HFD + DHEA + EX) group that received HFD with DHEA for 3 weeks and another 3 weeks of enoxacin (100 mg/kg/day) by intraperitoneal injection. Vaginal smears were performed respectively for 8 days before the end of modeling and treatment. Glucose Tolerance Test (GTT) and Insulin Tolerance Test (ITT) were performed respectively on day 35 and day 40. Fertility assessments were performed at the end of modeling and treatment (Day 42). Besides a reproductive capacity test, all mice were anesthetized and euthanized at the end of drug treatment (Day 43). Blood samples, Ovary, adipose tissue, and intestinal contents were carefully collected for further studies.

### Vaginal smears and estrous cycle determination

The estrous cycle phase of mice fed with a chow diet or HFD was assessed by vaginal smears. Vaginal smears were taken daily at 09:00 from the 13th to the 21st day after the first day of modeling and treatment. The smear was examined under light microscope after stained by methylene blue staining (0.1%, Solaibao. Beijing, China). The stage of the estrous cycle was determined by vaginal cytology with: proestrus (P) characterized mainly by round, nucleated epithelial cells; estrus (E) enriched for cornified squamous epithelial cells, metestrus (M) consisting of epithelial cells and leukocytes; diestrus (D) enriched for a predominance of leukocytes.

### Fertility assessment

The fertility assessment in this study was performed as described in two researches (Yuan et al., 2016; Qi et al., 2019). Female mice fed with HFD were mated with males of proven fertility 1:1. Successful mating was confirmed after the formation of a vaginal plug was checked. Female mice were euthanized to examine implantation sites to confirm pregnancy on 10 days after identification of vaginal plug. The rest of the animals were allowed to undergo natural delivery to produce pups.

### Glucose tolerance test and insulin tolerance test

For GTT, mice fed with a chow diet or HFD were fasted overnight (12 h). D-glucose (1 g/kg body weight) was injected intraperitoneally and the blood glucose levels were measured through the tail vein before and 15, 30, 60, 90, and 120 min after the injection of D-glucose with a glucometer (Roche). For ITT, mice fed with a chow diet or HFD were fasted for 4 h and intraperitoneally injected with insulin (0.5 U/kg body weight). Similarly, the blood glucose levels were measured by tail vein at different time points.

### Hormone analysis

The blood sample was centrifuged and serum was collected and stored at −80°C. The levels of testosterone (T), luteinizing hormone (LH), follicle stimulating hormone (FSH), and insulin in serum were measured by ELISA kit (CusaBio, Wuhan, China). The protocols were followed the manufacturer’s instructions.

### Hematoxylin-eosin staining

After fixation with 4% paraformaldehyde for 4h, adipose tissue and ovaries of mice were dehydrated, and embedded in paraffin. Paraffin blocks were sectioned into 4–5 μm thick sections. Then, sections were dewaxed and stained with hematoxylin and eosin, and examined under a microscope equipped with a DFC350FX digital camera (Leica, Milano, Italy). For immunohistochemical staining, deparaffinized sections were rehydrated and boiled in 10 mM citric acid for antigen retrieval, then incubated with primary antibody at 4°C overnight. The next day, all sections were incubated with secondary antibodies for 1 h at room temperature. 3,3′-diaminobenzidine (DAB) was selected as a chromogen to reveal the reaction. All these histological sections were visualized by using a light microscope (Leica, Milano, Italy).

### Western-blot analysis

Small pieces of adipose tissues were homogenized with lysis buffer. Total protein concentrations were determined by Bradford protein assay kit (Thermo Scientific, United States). Equal amounts of protein samples were loaded. Proteins were separated *via* 10% SDS-polyacrylamide gel electrophoresis (SDS-PAGE). Proteins were then transferred onto a polyvinylidene difluoride membrane (PVDF, Millipore, Billerica, MA, United States). Membranes were blocked in 5% skim milk for 60 min, Primary antibody (UCP-1, ab10983, Abcam, 1:2000 dilution; PGC-1α, ET1702-96, HUABIO, 1:2000 dilution) was then incubated overnight at 4°C. The next day membranes were washed and incubated in secondary antibody for 1 h. The target bands were detected by ECL chromogenic substrate. Protein bands were quantified using ImageJ software. All representative images were repeated in three independent experiments.

### Quantitative real-time PCR

Total mRNA of different tissues from female mice was isolated using TRIzol reagent (Invitrogen, Carlsbad, CA, United States) according to the manufacturer’s protocol. 2 μg of reverse transcription of total RNA was performed with the QuantiTect^®^ Reverse Transcription Kit (Takara Ltd., Otsu, Japan). Quantitative Realtime PCR using SYBR Green Realtime PCR Maser Mix (Vazyme, Nanjing, China) was performed to detect mRNA expression levels of target genes according to the manufacturer’s instructions. The amplification thermal cycling program was as follows: 3 min of initial denaturation at 95°C, then 40 cycles at 95°C for 30 s, 50°C for 30 s, 68°C for 1 min 30 s. Primer sequences are listed in Supplementary Table S1.

### *16S* rDNA sequencing

The fresh feces of mice fed with a chow diet were individually collected at the end of the administration period. Total DNA from the mice’s feces was extracted by QIAamp DNA fecal Mini Kit 206 according to the manufacturer’s instructions. The V3-V4 region of *16S* rDNA genes was amplified by PCR and extracted from 2% agarose gel, then purified by VAHTS DNA Clean beads. Then the *16S* rDNA gene amplicons were subsequently sequenced on Illumina Nova platform. The sequence data was analyzed using QIIME software (v1.8.0).

### Statistical analysis

All statistical analyses were performed with GraphPad (Prism 8.4.2). Data are expressed as mean ± SEM. The statistical significance was assessed using unpaired Student’s *t*-test (for two groups) or one-way ANOVA followed with Bonferroni’s post hoc test (for multiple groups). Normality and homogeneity of variance were evaluated with Kolmogorov-Smirnov analyses. Values *p* < *0.05* were considered significant (*p ≤ *0.05*, **p ≤ 0.01).

## Results

### Enoxacin treatment ameliorated irregular estrous cyclicity in dehydroepiandrosterone-induced polycystic ovary syndrome upon chow diet

**FIGURE 1:**
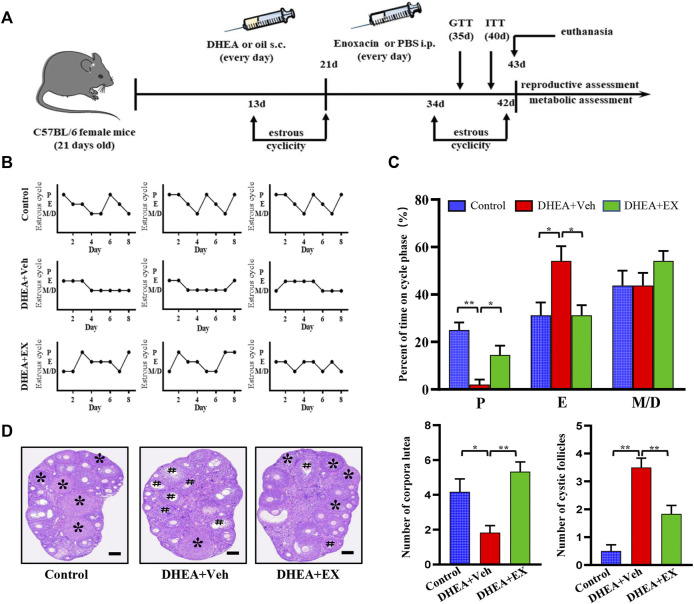
Enoxacin treatment ameliorated irregular estrous cyclicity in DHEA-induced PCOS upon chow diet. **(A)**Schematic of animal experiment process; **(B,C)** The estrous cycle of mice under chow diet conditions; **(D)** H&E staining of representative ovaries. The cystic follicle is indicated by a hashtag, while the corpora lutea are indicated by asterisks. Scale bar: 200 μm. Values are represented as the mean ± SE. *n* = 5–6. *P* values were determined by one-way ANOVA with Tukey’s multiple comparison post-hoc test. *p < 0.05, **p < 0.01. DHEA, dehydroepiandrosterone; Veh, vehicle; EX, enoxacin.

In order to verify the therapeutic effect of enoxacin on hyperandrogenia-related PCOS, enoxacin was injected to DHEA-induced PCOS mice subcutaneously. The reproductive function of the mice was evaluated by daily vaginal smear examination of estrus cycle (Figure 1A). As shown, the estrus cycle of DHEA-vehicle mice was disrupted and characterized by a continuous estrus period, and which in control group and enoxacin group was normal, ranging from 4 to 5 days (Figure 1B). In addition, the percentage of the estrus cycle at different stages was quantitatively analyzed. The results showed that the disrupted estrus cycle induced by DHEA could be improved after enoxacin treatment (Figure 1C). We then examined the morphology of the ovaries and found typical PCOS changes were observed in DHEA-vehicle group, including the appearance of cystic follicles and decreased number of luteum, however, these abnormities were abolished by enoxacin treatment (Figure 1D). In conclusion, enoxacin therapy can improve ovarian function in PCOS mice, including the interrupted estrus cycle, and abnormal ovarian morphology.

### Enoxacin treatment improved hormone disorders in dehydroepiandrosterone-treated mice upon chow diet

**FIGURE 2:**
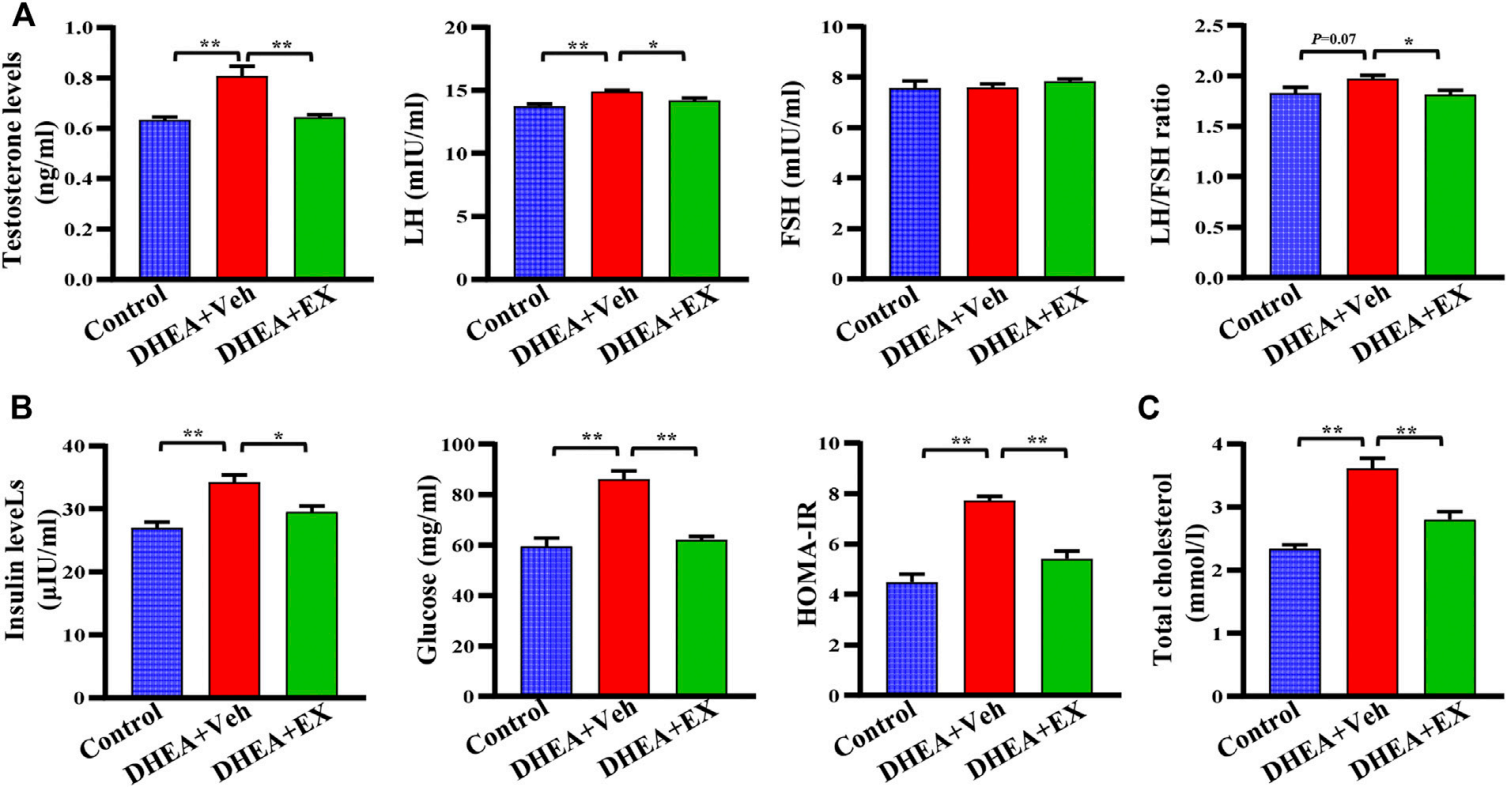
Enoxacin treatment improved hormone disorders in DHEA-treated mice upon chow diet. **(A)** The levels of reproductive hormones including testosterone, the LH, FSH and LH/FSH ratio. **(B)** The levels of insulin, fasting serum glucose, and HOMA-IR score. **(C)** Levels of serum total cholesterol. Values are represented as the mean ± SE. *n* = 5–6. *P* values were determined by one-way ANOVA with Tukey’s multiple comparison post-hoc test. *p < 0.05, **p < 0.01. DHEA, dehydroepiandrosterone; Veh, vehicle; EX, enoxacin.

It is well documented that hormonal disorders such as hyperandrogenemia are important features of PCOS. We next determined the levels of reproductive hormones, including testosterone, LH, and FSH. After enoxacin administration, the levels of serum testosterone and LH in DHEA-vehicle group were decreased, while no difference was observed in FSH levels (Figure 2A). Notably, the DHEA-induced increased LH/FSH ratio, a useful marker of ovarian reserves (Mukherjee et al., 1996), was also abolished in the enoxacin group (Figure 2A). Next, we observed that enoxacin-treated mice showed decreased serum insulin levels and HOMA-IR scores compared with mice in the vehicle group, further confirming the improvement of enoxacin on hyperinsulinemia (Figure 2B). Meanwhile, increased serum cholesterol levels in PCOS were also decreased by enoxacin (Figure 2C). Taken together, these results indicate that enoxacin treatment could improve hormone disorders including hyperandrogenism and hyperinsulinemia in DHEA-induced PCOS.

### Enoxacin treatment ameliorated irregular estrous cyclicity in dehydroepiandrosterone-induced polycystic ovary syndrome upon high-fat diet

**FIGURE 3:**
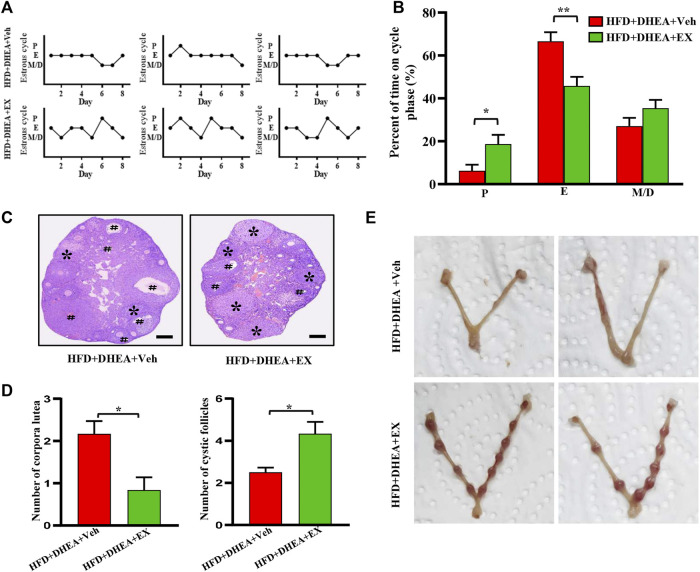
Enoxacin treatment ameliorated irregular estrous cyclicity in DHEA-induced PCOS upon HFD. **(A,B)** The estrous cycle of mice under HFD conditions; **(C,D)** H&E staining of representative mice ovaries under HFD conditions. The cystic follicle is indicated by a hashtag, while the corpora lutea are indicated by asterisks. Scale bar: 200 μm; **(E)** Representative images of embryo. Values are represented as the mean ± SE. *n* = 5–6. *P* values were determined by two-tailed Student’s *t*-test. *p < 0.05, **p < 0.01. DHEA, dehydroepiandrosterone; Veh, vehicle; EX, enoxacin.

It is well documented that obesity and PCOS are interrelated, obesity increases the prevalence of PCOS and PCOS results in weight gain and obesity. The PCOS women with obesity have a more severe phenotype compared to non-obese PCOS (Lim et al., 2013; Behboudi-Gandevani et al., 2016; Liu et al., 2017b). Thus, our study investigated the therapeutic effects of enoxacin on PCOS under HFD conditions. Consistently, compared with mice on vehicle, mice treated with enoxacin displayed a restored estrous cycle (Figures 3A,B). The histopathological analysis revealed follicles at different stages of development in enoxacin-treated mice towards normalization in numbers of corpora lutea (Figures 3C,D). To further investigate the therapeutical effect of enoxacin on embryo implantation in PCOS, fertility tests were performed by counting the number of embryos after mating (10 days after vaginal plug formation). Administration of enoxacin remarkably increased the number of embryos compared with vehicle-treated mice (Figure 3E). Taken together, these results indicate that enoxacin could help to modulate ovarian functions in the PCOS mouse model on HFD.

### Enoxacin treatment ameliorated insulin resistance in polycystic ovary syndrome

**FIGURE 4:**
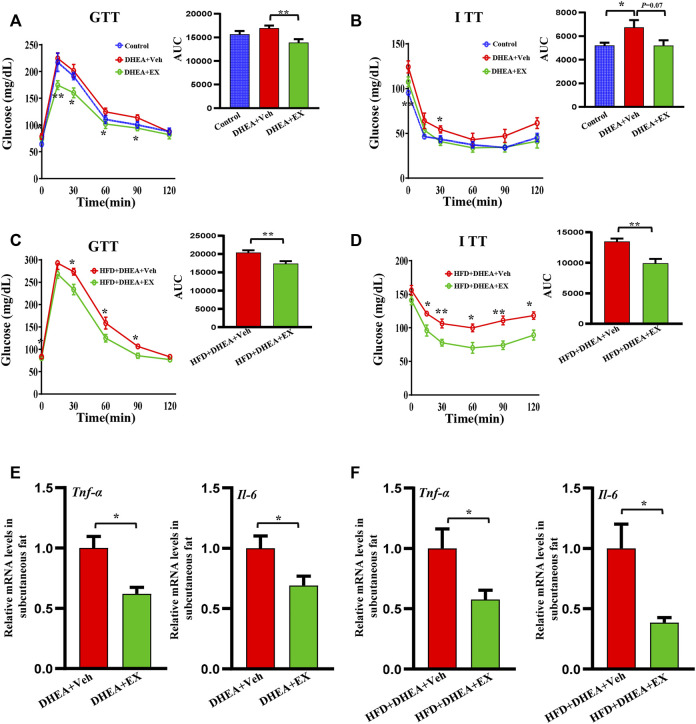
Enoxacin treatment ameliorated insulin resistance in PCOS. **(A,B)** GTT and ITT of mice upon chow diet conditions; **(C,D)** GTT and ITT of mice upon HFD conditions; **(E)** mRNA expression of inflammatory cytokines including *Tnf-α*, *Il-6* in upon chow diet conditions; **(F)** mRNA expression of inflammatory cytokines including *Tnf-α*, *Il-6* in upon HFD conditions. Values are represented as the mean ± SE. *n* = 5–6. For **(A,B)**, *p* values were determined by one-way ANOVA with Tukey’s multiple comparison post-hoc test. For **(C–F)**, *p* values were determined by two-tailed Student’s *t*-test. *p < 0.05, **p < 0.01. DHEA, dehydroepiandrosterone; Veh, vehicle; EX, enoxacin.

Regarding the role of insulin resistance in PCOS, we performed GTT and ITT analysis to detected the effect of enoxacin on glucose tolerance and insulin sensitivity. Compared with control mice, the PCOS mouse model displayed apparent insulin resistance, and enoxacin treatment restored this impairment (Figures 4A,B). Under HFD conditions, enoxacin also showed beneficial effects on glucose tolerance and insulin sensitivity in the PCOS mouse model (Figures 4C,D). Adipose tissue inflammation plays an essential role in the development of insulin resistance and PCOS (Delitala et al., 2017). In this study, mRNA expression of inflammatory cytokines *Tnf-α* and *Il-6* in subcutaneous fat were significantly decreased by enoxacin administration (Figures 4E,F). These results demonstrate that enoxacin attenuates insulin resistance and adipose inflammation in the PCOS mouse model.

### Enoxacin treatment promoted the browning of white adipose tissue in polycystic ovary syndrome

**FIGURE 5:**
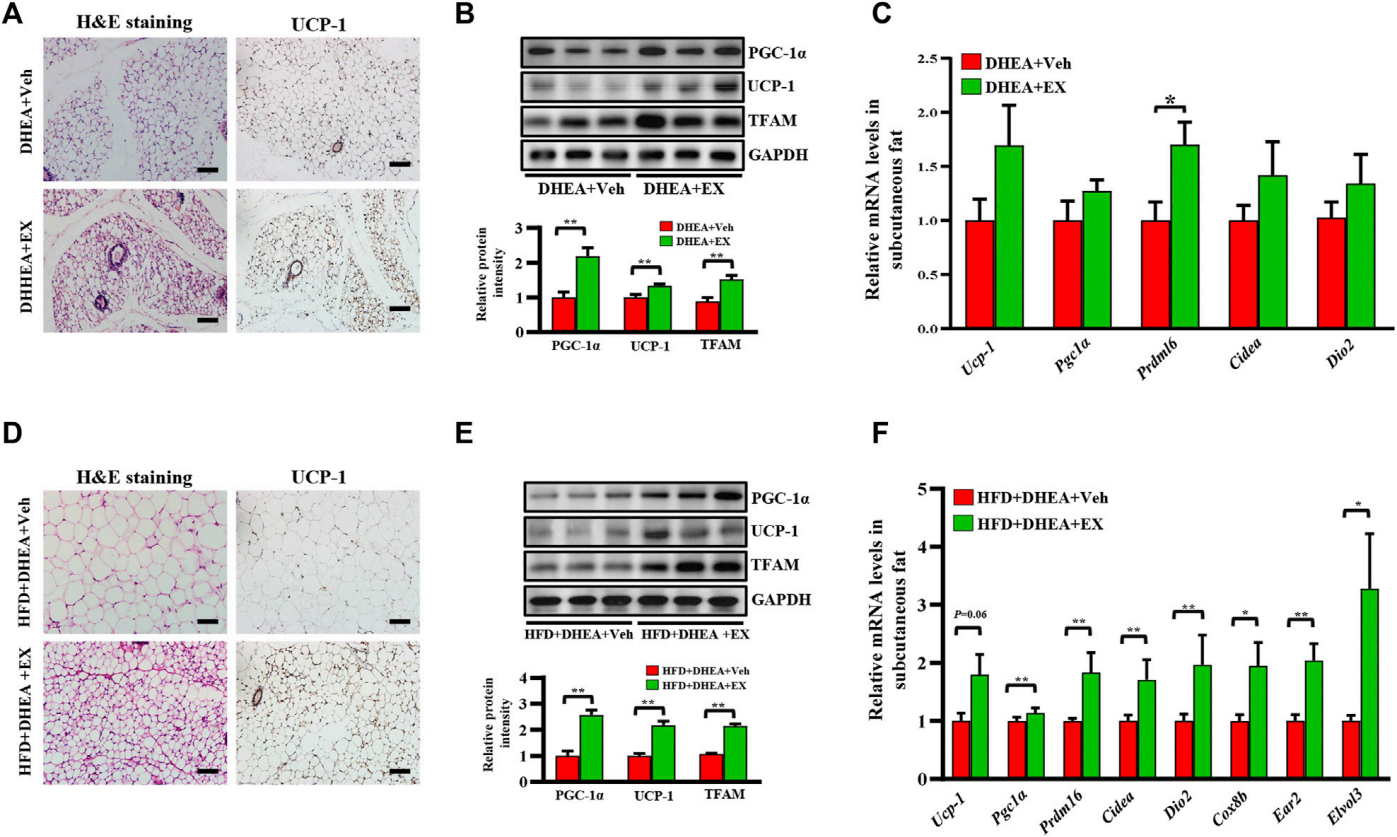
Enoxacin treatment promote the browning of white adipose tissue in PCOS. **(A)** H&E staining and UCP-1 immunohistochemical staining of subcutaneous adipose tissue under chow diet conditions. Scale bar: 100 μm; **(B)** Western analysis of PGC-1α, UCP-1 and TFAM in subcutaneous adipose tissue upon chow diet. Relative intensity was calculated by using ImageJ software. **(C)** mRNA expression of thermogenic genes of subcutaneous adipose tissue upon chow diet; **(D)** H&E staining and UCP-1 immunohistochemical staining of subcutaneous adipose tissue under HFD. Scale bar: 100 μm; **(E)** Western analysis of PGC-1α, UCP-1 and TFAM in subcutaneous adipose tissue under HFD. Relative intensity was calculated by using ImageJ software; **(F)** mRNA expression of thermogenic genes of subcutaneous adipose tissue under HFD. Values are represented as the mean ± SE. *n* = 5–6. *P* values were determined by two-tailed Student’s *t*-test. *p < 0.05, **p < 0.01. DHEA, dehydroepiandrosterone; Veh, vehicle; EX, enoxacin.

PCOS has been reported to be associated with white fat dysfunction, WAT browning has been previously demonstrated to increase the metabolic rate and insulin sensitivity, and has a therapeutic role in PCOS (Zhang et al., 2022). Therefore, we performed a series of experiments to evaluate the effect of enoxacin on white fat in PCOS. As shown in Supplementary Figures S1, S2, weight of adipose tissue and ratio of adipose tissue weight/body weight were shown to be reduced in enoxacin-treated mice under both chow diet and HFD conditions (Supplementary Figures S1, S2). Furthermore, compared with the DHEA-vehicle group, smaller adipocyte area and more multilocular beige adipocytes were observed in subcutaneous fat from enoxacin-treated mice (Figure 5A). Thus, we speculated that enoxacin intervention may exert an impact on lipid metabolism. As shown in immumohistochemical staining, the expression of UCP-1 (an indicator of thermogenesis (Wu et al., 2012) was increased by enoxacin treatment in white fat (Figure 5A). Consistently, Western blot analysis further validated that enoxacin enhanced the expression of UCP-1, as well as the master mitochondrial regulators PGC-1α and TFAM (Figure 5B). Meanwhile, qRT-PCR analysis revealed the upregulated thermogenic gene such as *Prdm16* in enoxacin-treated mice (Figure 5C). Consistently, mice in enoxacin-treated group on HFD also displayed an increased number of beige adipocytes and enhanced expression of thermogenic factors in subcutaneous adipose tissue (Figures 5D–F). These results demonstrate that enoxacin treatment promotes the browning of adipose tissue and increases thermogenesis in PCOS.

### Enoxacin modified the structure and composition of the gut microbiota in polycystic ovary syndrome

**FIGURE 6:**
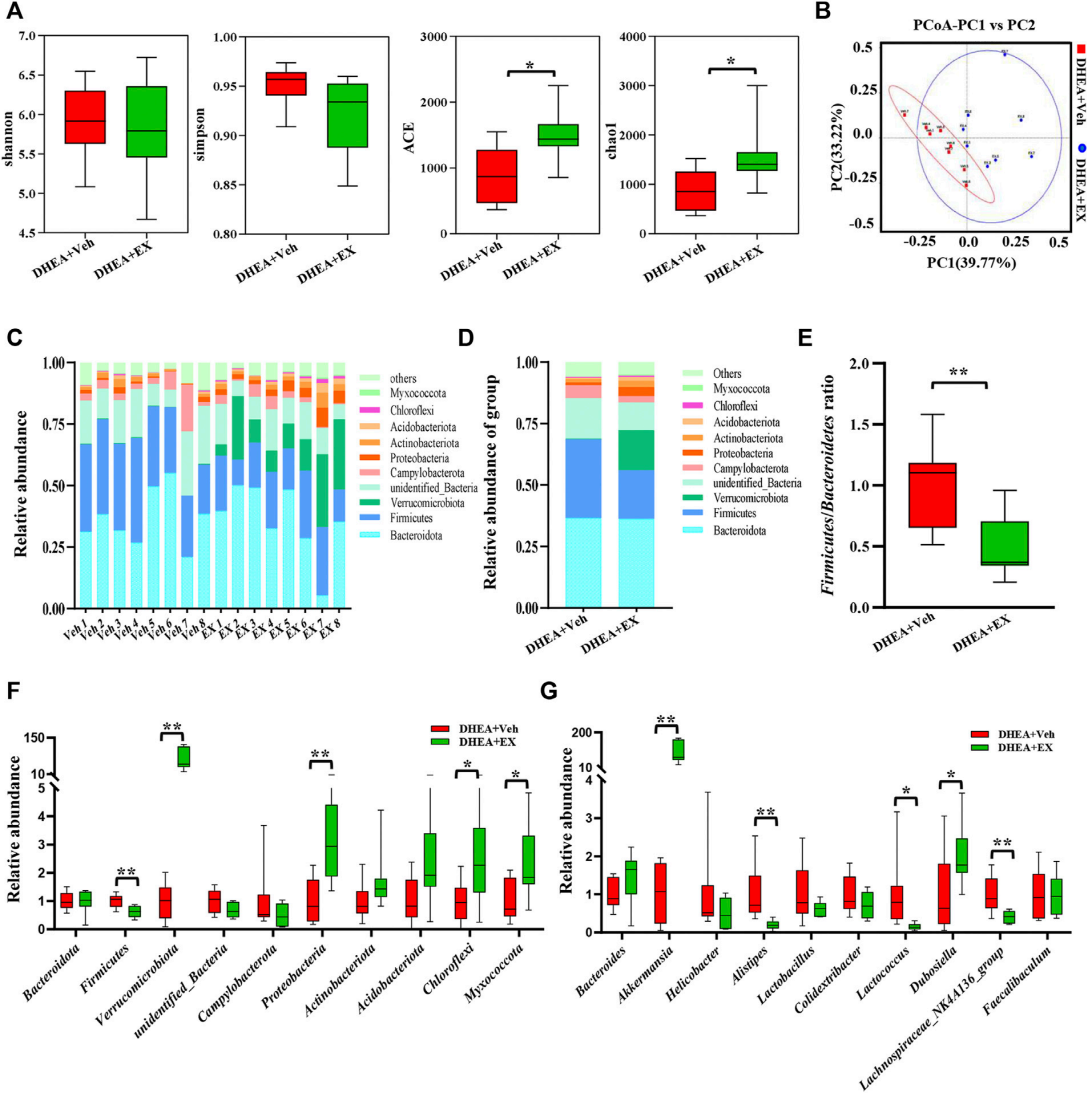
Enoxacin treatment modified the structure and composition of the gut microbiota in PCOS. **(A)**α-diversity of the gut microbiota between the vehicle and enoxacin groups, as indicated by the Shannon, Simpson ACE, and Chao1 indices. **(B)** Principal Coordinate Analysis (PCoA) based on the Bray Curtis distance algorithm. **(C)**Taxonomic composition of each mouse at the phylum level. **(D)** Taxonomic composition of two group at the phylum level. **(E)** Firmicutes/Bacteroidetes ratio. **(F)** Relative abundance of gut microbiota on phylum level. **(G)** Relative abundance of gut microbiota on genus level. Values are represented as the mean ± SE. *n* = 5–6. *P* values were determined by two-tailed Student’s *t*-test. *p < 0.05, **p < 0.01. DHEA, dehydroepiandrosterone; Veh, vehicle; EX, enoxacin.

Recently, the importance of gut microbiota in host energy metabolism has been widely acknowledged, dysbiosis of gut microbiota leads to multiple metabolic diseases including PCOS (Crunkhorn, 2019). In this study, high-throughput sequencing technology is used to target *16S* rDNA genes to determine whether enoxacin caused changes in the structure and composition of gut microbiota. Abundance-based coverage estimator (ACE) and Chao1 indices indicated that there was a significant difference between the two groups in *α* diversity (Figure 6A). The *β* diversity showed that the composition of the gut microbiota was markedly altered upon enoxacin administration (Figure 6B). We examined the changes at the phylum level and found that the abundance of *Proteobacteria* and *Verrucomicrobiota* phylum members was higher, whereas the abundance of *Firmicutes* phylum members was lower, in enoxacin- versus vehicle-treated mice (Figure 6C). The increase in *Firmicutes* or decrease in *Bacteroidetes* counts is generally considered to be indicative of a dysbiosis of the gut microbiota, which has been associated with obesity, diabetes, and cardiovascular disease (Santos-Marcos et al., 2019). Our study revealed a significantly reduced *Firmicutes*/*Bacteroidetes* (*F/B*) ratio in enoxacin-treated mice compared with controls (Figures 6D,E). Quantitative analysis of gut microbiota indicated that apart from *Firmicutes* and *Proteobacteria*, the abundance of *Chloroflexi* and *Myxococcota* was increased by enoxacin on the phylum level (Figure 6F). Meanwhile, the abundance of genus *Akkermansia* spp. and *Dubosiella* spp. was higher, whereas the abundance of genus *Alistipes, Lactococcus* and *Lachnospiraceae_NK4A136* members was clearly lower in enoxacin- versus vehicle-treated mice (Figure 6G).

**FIGURE 7:**
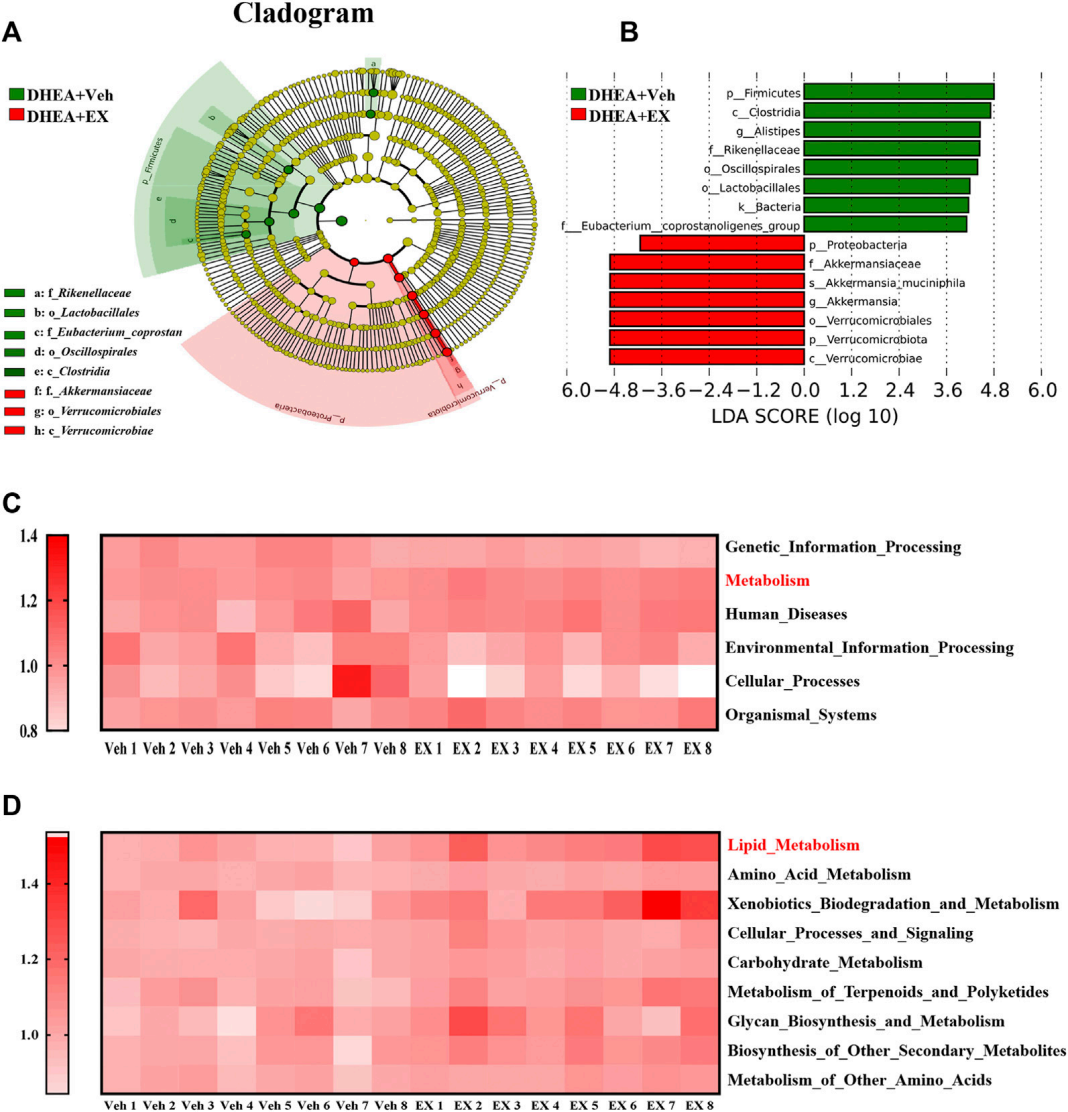
Gut microbiota comparisons between the two groups analyzed by LEfSe and KEGG. **(A,B)** Taxonomic cladogram generated from LEfSe of metagenomic sequencing data. Green indicates enriched taxa in the vehicle group. Red indicates enriched taxa in the enoxacin group. Each circle’s size is proportional to the taxon’s abundance. **(C,D)** Kyoto Encyclopedia of Genes and Genomes annotation (KEGG) of key metabolic pathways at different levels. Values are represented as the mean ± SE. *n* = 5–6. *P* values were determined by two-tailed Student’s *t-*test. *p < 0.05, **p < 0.01. DHEA, dehydroepiandrosterone; Veh, vehicle; EX, Enoxacin.

The differences of abundant bacterial taxa between the two groups were performed by Linear discriminant analysis Effect Size (LEfSe) analysis. As shown in Figures 7A,B **,** mice in the enoxacin-treated group were characterized by phylum *Verrucomicrobiae* and *Proteobacteria*, and related species such as *Akkermansia muciniphila*. Whereas several microbes belonging to the *Firmicutes*, such as *Alistipes*, were considered as the key genus in the vehicle-treated group (Figures 7A,B). Subsequently, KEGG and GO analyses were performed to predict the potential function and explore the underlying mechanisms. Notably, enoxacin treatment enriched KEGG pathways involved in metabolism, especially the lipid metabolism, compared to the DHEA-vehicle mice (Figures 7C,D). These results indicate that enoxacin modified the structure and composition of the gut microbiota in PCOS.

## Discussion

PCOS is a metabolic disease of endocrine disorders in women, and fat metabolism has been confirmed to be an effective therapeutic target (Zhang et al., 2022). Brown adipose tissue transplantation can significantly improve metabolic symptoms and reproductive disorders, however, the operational feasibility is poor (Yuan et al., 2016). Thermogenesis through exogenous activation, such as treatments of certain plant extracts (e.g., Rutin), cytokines (e.g., IL-22), may provide an alternative option (Hu et al., 2017; Qi et al., 2019). A recent study has found that enoxacin induces thermogenesis and prevents obesity by regulating miR-34a-5p expression in adipose tissue (Rocha et al., 2020). However, it is unknown whether these functions exist in DHEA-induced PCOS model and achieve the desired therapeutic effect of ameliorating reproductive and endocrine dysfunction. Excitingly, our results showed that the disrupted estrus cycle, abnormal hormone levels and morphology of the ovaries induced by DHEA had been partly recovered by enoxacin. Consistently, enoxacin did upregulate the expression of some thermogenic regulators in white fat, especially in the obese PCOS mouse model. These findings further confirmed that reproductive disorders in PCOS can be ameliorated by activating fat thermogenesis.

Strong evidence indicated that gut microbiome has emerged as a critical regulator of host metabolism, and the glucose and lipid metabolic disorders are often accompanied by gut dysbiosis (Cani et al., 2019). Meanwhile exposure to cold condition induces changes in the composition of the gut microbiota, while faecal transplantation from cold-exposed donors was shown to replicate the lean and prothermogenic phenotype in recipient mice independent of diet (Chevalier et al., 2015; Ziętak et al., 2017). The regulation of energy metabolism by gut microbiome is mainly mediated by specific bacterial groups or its metabolites. Among them, the effects of the *Akkermansia* on energy metabolism have been widely investigated. Two studies revealed that *Akkermansia* treatment led to decreased energy efficiency and enhanced WAT browning in HFD-fed mice (Schneeberger et al., 2015; Gao et al., 2018). Similarly, in HFD-fed mice supplemented with tea or coffee extracts, the higher abundance of *Akkermansia* was positively correlated with lipid oxidation and browning processes (Liu et al., 2017a; Gao et al., 2018). In addition, microbial metabolites such as short-chain fatty acids, bile acids, indole propionic acid, branched chain amino acids and endocannabinoids can also promote thermogenesis and WAT browning *via* direct or indirect mechanisms (Cani et al., 2019). In this study, we found the abundance of *Akkermansia* was increased by enoxacin, which might be, at least partly, involved in the regulation of the browning of white fat.

The pathophysiology of metabolic disease induced by obesity is closely linked to WAT dysfunction through mechanisms including oxidative stress, apoptosis, fibrosis, inflammation, and mitochondrial dysfunction (Furukawa et al., 2004; Crewe et al., 2017; Saltiel and Olefsky, 2017; Heinonen et al., 2020). Mitochondria are essential for maintaining metabolic homeostasis in white adipocytes for their involvement in ATP production, fatty acid synthesis and oxidation. Mitochondrial content and function were shown to be decreased in WAT of obese humans and animals (Wilson-Fritch et al., 2004; Choo et al., 2006; Mustelin et al., 2008). Impaired oxidative capacity produces excess reactive oxygen species (ROS), leading to increased inflammation in adipose tissue (Heinonen et al., 2020). Furthermore, multiple studies have reported an increase in mitochondrial mass and function in WAT of diet-induced obesity in the progress of WAT browning (Butow and Bahassi, 1999; Gao and Houtkooper, 2014; Yu and Pekkurnaz, 2018), which further confirms the key role of mitochondria in energy metabolism. In the present study, enoxacin was shown to increase the expression of the master mitochondrial regulators PGC-1α and TFAM. Thus, we postulate that enoxacin might promote mitochondrial function or biogenesis in white adipose tissue and provide metabolic benefits in PCOS.

Enoxacin is a broad-spectrum fluoroquinolone antibacterial agent, but shown to increase the diversity of gut microbiota in PCOS mice in this study. A recent study has found that intraperitoneal injection of enoxacin does not affect the bacterial community structure under normal conditions (Rocha et al., 2020). Meanwhile, patients with PCOS have a lower diversity and an altered phylogenetic profile in the microbiome (Liu et al., 2017b; Lindheim et al., 2017; Torres et al., 2018). Thus, we postulate that enoxacin treatment restores gut dysbiosis and thereby contributes to normalize the diversity of gut microbiota. Moreover, the third generation of fluoroquinolone antibacterial drugs such as norfloxacin, pefloxacin, and ciprofloxacin have been shown to influence the gut microbiome (Vollaard et al., 1990; Haak et al., 2019; Li et al., 2021). However, whether these drugs have beneficial effects on glucose and lipid metabolism has not been reported. By contrast, enoxacin has been shown to ameliorate diet-induced obesity by inducing white-fat browning in previous research (Rocha et al., 2020). In this study, we found that enoxacin can improve PCOS by improving fat metabolism and restoring gut dysbiosis, which further confirmed that the effect of enoxacin on glucose and lipid metabolism might be independent of its own antibacterial activity.

These studies have several potential limitations. First, adipose tissue has been identified as a secretory organ for adipokines, such as adiponectin, leptin, and resistin, which are involved in the pathogenesis of PCOS (Mitchell et al., 2005; Behboudi-Gandevani et al., 2016). Adiponectin is the strongest factor associated with insulin resistance in PCOS, previous studies have shown that plasma adiponectin concentrations are lower in women with PCOS (Sepilian and Nagamani, 2005). Increased secretion of adiponectin can mitigate prepubertal androgen-induced metabolic disorders in dihydrotestosterone-exposed mice with PCOS-like phenotypes (Benrick et al., 2017). However, whether adipokines such as adiponectin would participant in the regulation of enoxacin in PCOS remain to be determined. Second, the gut microbiota may affect the host metabolism, immunity, and health through its metabolites such as short-chain fatty acids, and bile acids (Shapiro et al., 2018; Pei Luo et al., 2022). Whether changes in microbial composition induced by enoxacin are accompanied by changes in metabolites and the impact on PCOS remains to be determined. Finally, in addition to DHEA, many agents such as letrozole, dihydrotestosterone and sodium prasterone sulfate have been widely used to establish PCOS animal models (Stener-Victorin et al., 2020). Recently, it has been reported that a mouse model of prenatal anti-Mullerian hormone (PAMH) has neuroendocrine characteristics similar to those of individuals with PCOS (Tata et al., 2018). Most of these models have the key features of polycystic ovary, but can only reflect one or several aspects of the characteristics of PCOS in human. Therefore, it is necessary to construct other animal models to evaluate the effect of enoxacin on PCOS.

## Conclusion

In summary, enoxacin ameliorated reproductive disorders and insulin resistance of DHEA-induced PCOS by promoting the browning of white fat and restoring gut dysbiosis. These findings provide a novel therapeutic target of enoxacin in PCOS clinical application.

## Data Availability

The datasets presented in this study can be found in online repositories. The names of the repository/repositories and accession number(s) can be found below: https://www.ncbi.nlm.nih.gov; PRJNA853866.

## Glossary

PCOS: Polycystic ovary syndrome
IR: Insulin resistance
DHEA: Dehydroepiandrosterone
HFD: High fat diet
RT-PCR: Reverse transcription-polymerase chain reaction analyses
PBS: Phosphate-buffered saline
GTT: Glucose tolerance test
ITT: Insulin tolerance test
ELISA: Enzyme linked immunosorbent assay
T: Testosterone
LH: Luteinizing hormone
FSH: Follicle stimulating hormone
SDS-PAGE: SDS-polyacrylamide gel electrophoresis
PVDF: Polyvinylidene difluoride membrane
UCP-1: Uncouple protein-1
PGC-1α: Peroxisome proliferator-activated receptor-gamma coactivator-1α
HOMA-IR: Homeostatic model assessment of insulin resistance
Dio 2: Iodothyronine deiodinase2
Tnf-α: Tumor necrosis factor-α
Il-6: Interleukin-6
Prdm16: Positive regulatory domain zinc finger region protein 16
PCOA: Principal coordinates analysis
LDA: Linear discriminant analysis
LEfSe: Linear discriminant analysis effect size
KEGG: Kyoto Encyclopedia of Genes and Genomes annotation
FGF21: Fibroblast growth factor 21
FMT: Fecal microbiota transplants
PAMH: Prenatal anti-Mullerian hormone
EX: Enoxacin
H&E staining: Hematoxylin-eosin staining
