# Supplementary material for: Enoxacin ameliorates polycystic ovary syndrome by promoting the browning of white adipose tissue and restoring gut dysbiosis
Source: Front Pharmacol. 2022 Sep 6;13:978019. doi: 10.3389/fphar.2022.978019 (PMC9486322; doi:10.3389/fphar.2022.978019)
Supplement: Supplementary file 1 [file DataSheet1.docx]

**Supplementary information**

**Table S1. List of primers sequences for qRT-PCR analysis**

| **Gene** | **Fwd (5’-3’)** | **Rev (5’-3’)** |
| --- | --- | --- |
| ***mUcp-1*** | TCTCAGCCGGCTTAATGACTG | GGCTTGCATTCTGACCTTCAC |
| ***m******Pgc-1α*** | GCACCAGAAAACAGCTCCAAG | CGTCAAACACAGCTTGACAGC |
| ***mPrdm16*** | ACACGCCAGTTCTCCAACCTGT | TGCTTGTTGAGGGAGGAGGTA |
| ***mCidea*** | TCCTATGCTGCACAGATGACG | TGCTCTTCTGTATCGCCCAGT |
| ***mCited1*** | AACCTTGGAGTGAAGGATCGC | GTAGGAGAGCCTATTGGAGATGT |
| ***mDio2*** | CATTGATGAGGCTCACCCTTC | GGTTCCGGTGCTTCTTAACCT |
| ***mCox8b*** | TGCTGGAACCATGAAGCCAAC | AGCCAGCCAAAACTCCCACTT |
| ***mElvol3*** | GTGTGCTTTGCCATCTACACG | CTCCCAGTTCAACAACCTTGC |
| ***mTnf-α*** | GGCGGTGCCTATGTCTCA | AGGGTCTGGGCCATAGAA |
| ***mIl-6*** | TTCTTGGGACTGATGCTG | CTCATTTCCACGATTTCCC |
| ***18s*** | TTGACTCAACACGGGAAACC | AGACAAATCGCTCCACCAAC |

**Supplementary Figure 1** (A)Weight of white adipose tissue(g) in mice on chow diet. (B) Ratio of WAT weight/body weight(%) in mice on chow diet .Values are represented as the mean ± SE. n = 6. *p* values were determinted by one-way ANOVA with Tukey’s multiple comparison post-hoc test. ∗*p* <0.05, ∗∗*p* <0.01. DHEA, dehydroepiandrosterone; Veh, vehicle; EX, enoxacin

**Supplementary Figure 2** (A)Weight of white adipose tissue(g) in mice on HFD. (B) Ratio of WAT weight/body weight(%) in mice on HFD.Values are represented as the mean ± SE. n = 6. *p* values were determined by two-tailed Student’s *t*-test. ∗*p* <0.05, ∗∗*p* <0.01. DHEA, dehydroepiandrosterone; HFD, high fat diet; Veh, vehicle; EX, enoxacin
